# Using random forests to diagnose aviation turbulence

**DOI:** 10.1007/s10994-013-5346-7

**Published:** 2013-04-23

**Authors:** John K. Williams

**Affiliations:** Research Applications Laboratory, National Center for Atmospheric Research, P.O. Box 3000, Boulder, CO USA

**Keywords:** Turbulence, Aviation, Weather, Air traffic, Thunderstorms, Random forest, Data fusion

## Abstract

Atmospheric turbulence poses a significant hazard to aviation, with severe encounters costing airlines millions of dollars per year in compensation, aircraft damage, and delays due to required post-event inspections and repairs. Moreover, attempts to avoid turbulent airspace cause flight delays and en route deviations that increase air traffic controller workload, disrupt schedules of air crews and passengers and use extra fuel. For these reasons, the Federal Aviation Administration and the National Aeronautics and Space Administration have funded the development of automated turbulence detection, diagnosis and forecasting products. This paper describes a methodology for fusing data from diverse sources and producing a real-time diagnosis of turbulence associated with thunderstorms, a significant cause of weather delays and turbulence encounters that is not well-addressed by current turbulence forecasts. The data fusion algorithm is trained using a retrospective dataset that includes objective turbulence reports from commercial aircraft and collocated predictor data. It is evaluated on an independent test set using several performance metrics including receiver operating characteristic curves, which are used for FAA turbulence product evaluations prior to their deployment. A prototype implementation fuses data from Doppler radar, geostationary satellites, a lightning detection network and a numerical weather prediction model to produce deterministic and probabilistic turbulence assessments suitable for use by air traffic managers, dispatchers and pilots. The algorithm is scheduled to be operationally implemented at the National Weather Service’s Aviation Weather Center in 2014.

## Problem of interest

### Aviation impact of en-route turbulence

Nearly everyone who has flown in an aircraft has experienced atmospheric turbulence—that unnerving jolting up and down and side to side that feels like an amusement park ride gone bad. Although commercial airline crashes are now exceedingly rare, turbulence encounters continue to remind passengers that flying is not completely safe. Indeed, according to a study of National Transportation Safety Board (NTSB) data from 1983–1997, turbulence was the predominant cause of accidents and injuries in large commercial aircraft, accounting for about 70 % of all weather-related incidents (Eichenbaum 2000). Including general aviation (GA) aircraft, turbulence contributed to 664 accidents leading to 609 fatalities (all but 3 GA), 239 serious injuries and 584 minor injuries over that time period. A joint government and industry study (CAST 2001) estimated that there were 15,000 minor injuries on commercial aircraft over the same period that were not reported to the NTSB. The CAST study also found that 13 % of NTSB cases included minor cabin damage, and 4 % were associated with severe aircraft damage. One study estimated the costs to U.S. airlines associated with these incidents, including liability lawsuits, lost crew work time, inspections, and repairs at nearly $200 million per year (Eichenbaum 2003). Moreover, even turbulence that doesn’t cause injuries or accidents shapes the public’s impression of an airline’s safety, thus affecting its business. Sharman et al. (2006a) note that pilot reports (PIREPs) of moderate-or-greater turbulence encounters average about 65,000 per year, and severe-or-greater reports average 5,500 per year.

Airline dispatchers and pilots strive to avoid turbulence or quickly exit turbulent regions when they chance upon them. A severe or extreme turbulence report frequently causes the aviation weather forecaster responsible for that region to issue a turbulence Significant Meteorological Information advisory (SIGMET), and the airspace nearby can be shut down to air traffic or have its capacity for flights reduced. Pilots’ requests for route changes to find smoother air can significantly increase air traffic controller workload; a survey of FAA Air Route Traffic Control Centers, Traffic Management Units and Terminal Radar Control (Cook 2008) found that turbulence ranked behind only ceiling and visibility as the most important weather factor affecting the National Air Space (NAS). Avoiding areas of suspected turbulence results in increased fuel expenditure and flight delays that can cascade into disruptions to other flights and quickly escalating costs (Cook et al. 2004).

Atmospheric turbulence is often generated by large-scale forcing mechanisms such as the jet stream, upper level fronts, wind flow over rough terrain, or thunderstorm updrafts, downdrafts, windshear and outflow. Avoiding turbulence associated with thunderstorms, known as convectively-induced turbulence (CIT), is particularly difficult, and CIT has been estimated to be responsible for 60 % of turbulence-related aircraft accidents (Cornman and Carmichael 1993). 86 % of the 44 aviation accident cases analyzed by Kaplan et al. (2005) were found to be within 100 km of convection. CIT can exist at small scales and short time periods, and the dynamic evolution of thunderstorms makes CIT notoriously difficult to accurately diagnose or forecast (Lane et al. 2012).

### Limitations of thunderstorm avoidance guidelines

Thunderstorms are responsible for aviation hazards including lightning, hail, windshear, and airframe icing, but CIT is unique in that it can produce a threat well away from the active storm. For this reason, FAA guidelines specify that aircraft should circumnavigate thunderstorms by wide margins, affecting large regions of airspace on days of widespread convection. They include the following (Federal Aviation Administration 2012):

1. Don’t land or takeoff in the face of an approaching thunderstorm. A sudden gust front of low level turbulence could cause loss of control.
2. Don’t attempt to fly under a thunderstorm even if you can see through to the other side. Turbulence and wind shear under the storm could be disastrous.
3. Don’t fly without airborne radar into a cloud mass containing scattered embedded thunderstorms …
4. Don’t trust the visual appearance to be a reliable indicator of the turbulence inside a thunderstorm.
5. Do avoid by at least 20 miles any thunderstorm identified as severe or giving an intense radar echo. This is especially true under the anvil of a large cumulonimbus.
6. Do clear the top of a known or suspected severe thunderstorm by at least 1,000 feet altitude for each 10 knots of wind speed at the cloud top …
7. Do circumnavigate the entire area if the area has 6/10 thunderstorm coverage.
8. Do remember that vivid and frequent lightning indicates the probability of a strong thunderstorm.
9. Do regard as extremely hazardous any thunderstorm with tops 35,000 feet or higher …

Interpretation of these guidelines is subjective and limited by the information available to airline dispatchers and pilots. Moreover, recent studies have shown that significant CIT may occur outside these prescribed margins and may be dependent on environmental factors invisible to a pilot (Lane et al. 2012), while the NEXRAD Turbulence Detection Algorithm (NTDA) has frequently identified in-cloud areas near storms with benign null or light turbulence (Williams et al. 2006, 2008b). For these reasons, an automated, real-time gridded product that explicitly identifies hazardous regions of CIT is desirable.

### Limitations of operational turbulence forecasts

The study by Eichenbaum (2000) found that only 65 % of the NTSB turbulence-related accidents had either a valid Airman’s Meteorological Information advisory (AIRMET) or SIGMET at the time and location of the accident, and thus 35 % would have been unexpected by the flight crews. Moreover, AIRMET and SIGMET polygons are often relatively large compared to the actual hazard area. In order to improve the guidance available to dispatchers and air traffic managers, the FAA Aviation Weather Research Program (AWRP) has sponsored the development of the Graphical Turbulence Guidance (GTG) product (Sharman et al. 2006b). GTG uses operational rapid-update numerical weather prediction (NWP) model output to compute a number of derived turbulence “diagnostics” and combines them using fuzzy logic to create 3-D, 0–12 hour turbulence forecasts over the conterminous U.S. (CONUS). GTG runs operationally at the National Weather Service (NWS) Aviation Weather Center (AWC) and is available online via the Aviation Digital Data Service (ADDS) at aviationweather.gov/adds/turbulence/.

GTG development (Sharman et al. 2006a, 2006b) has followed an incremental approach, focusing first on upper-level clear-air turbulence (CAT) and then extending to mid-levels and addressing mountain-wave turbulence (MWT). However, the currently operational version, GTG 2.5, does not explicitly address CIT. Both GTG 2.5 and the new GTG 3 currently under development use the Weather Research and Forecasting Rapid Refresh (WRF-RAP) NWP model (Benjamin et al. 2006), which produces hourly 13-km horizontal resolution forecasts that are too coarse to fully resolve thunderstorms. Moreover, WRF-RAP forecasts are not available until an hour or more after they are initialized, and hence often do not provide meaningful information about new and rapidly evolving storms.

In recent years, analysis of en-route turbulence reports, case studies and fine-scale simulations have facilitated better understanding of some sources of CIT. These include the production, upward propagation and breaking of gravity waves when a convective cell penetrates the tropopause (Lane et al. 2003; Trier et al. 2012); however, conditions for wave breaking appear highly dependent on the storm environment (Lane and Sharman 2008). Fovell et al. (2007) showed that ducted gravity waves could propagate away from a storm and cause isolated, transient regions of turbulence. Trier and Sharman (2009) found that the outflow from large storm systems could interact with the ambient flow to cause wind shear that produced turbulence hundreds of km away and hours later. These studies suggest that combining information about the storm environment obtained from NWP models with much higher resolution remote observations of storm attributes will be necessary to best identify regions of likely CIT. However, doing so requires a more flexible data fusion methodology than that currently used by GTG.

### Need for tactical turbulence diagnosis products

The real-time information about the current turbulent state of the atmosphere required by pilots and dispatchers for making tactical en-route decisions is not adequately provided via the FAA’s thunderstorm avoidance guidelines or by currently operational turbulence forecasts. To address this deficiency, the FAA Aviation Weather Research Program (AWRP) has sponsored the development of a GTG “Nowcast” (GTGN) component for GTG 3 that will combine turbulence observations, inferences and forecasts to produce new turbulence assessments every 15 minutes. One component of the GTGN system is a Diagnosis of CIT (DCIT) module that focuses on identifying likely regions of existing near-storm turbulence based on the latest observations and NWP model data (Williams et al. 2008b, 2012). The DCIT and GTGN products will also address requirements for the Next Generation Air Transportation System (NextGen), which includes gridded deterministic and probabilistic nowcasts and forecasts to support the increasing automation of air traffic management systems (JPDO 2008; Lindholm et al. 2010; Sharman and Williams 2009).

## Data sources and preparation

The goal of DCIT is to fuse remote observations and NWP model data in a real-time algorithm to produce 3-D gridded diagnoses of CIT. The “truth” data used for DCIT training and evaluation must be objective and have sufficiently high temporal and spatial resolution to capture even small-scale, transient patches of CIT. The predictor data must be operational (i.e., reliable), free or low-cost and readily available at the AWC via standard communications channels with minimal latency.

### Automated aircraft turbulence reports

The FAA sponsored the development and implementation on select commercial aircraft of a system that provides high-resolution, automated, objective turbulence reports suitable for use as “truth” in training DCIT (Cornman et al. 1995, 2004). The system reports eddy dissipation rate (EDR, *ε* ^1/3^), an aircraft-independent atmospheric turbulence metric, including both the median and peak EDR over approximately one-minute intervals. DCIT’s prediction target is the peak EDR because it supplies a good indication of the turbulence hazard and is better distributed over the reporting bins than the median value.

NCAR maintains a database of these *in situ* EDR reports from approximately 95 United Airlines (UAL) Boeing 757 (B757) aircraft and approximately 80 Delta Air Lines (DAL) Boeing 737 (B737) aircraft. UAL data are primarily from routes between the northeast and southwest U.S., while DAL also includes the southeast and thus provides somewhat more comprehensive coverage. The UAL algorithm, developed in the mid-1990s, utilizes aircraft accelerometer data along with an aircraft response model. EDR data are coarsely binned with just 8 bins centered at 0.05, 0.15, 0.25, 0.35, 0.45, 0.55, 0.65 and 0.75 m^2/3^ s^−1^, time is reported to the nearest minute, time and position are given for every fourth report, and the algorithm’s onboard quality control capabilities are limited. The DAL implementation, developed several years later, utilizes aircraft-measured vertical winds, obviating the need for an aircraft response function, and includes a sophisticated on-board quality control algorithm. It bins the EDR data at 0.02 m^2/3^ s^−1^ and reports time to the nearest 10 s. However, in order to save communications costs, it does not transmit every report. Rather, it produces a routine report every 15 minutes and includes “triggered” reports when the measured turbulence reaches certain intensity or longevity thresholds. It does report all peak EDR≥0.20 m^2/3^ s^−1^.

*In situ* EDR report values may be roughly associated with atmospheric turbulence severity, although actual aircraft experience of turbulence depends on factors such as its weight, structure (particularly wing area), airspeed, and altitude. For instance, light aircraft generally experience more severe turbulence because they are more easily moved by variable winds. The International Civil Aviation Organization (2001) recommended interpreting 0.0–0.1 as null turbulence, 0.1–0.2 as light, 0.2–0.3 as light to moderate, 0.3–0.4 as moderate, 0.4–0.5 as moderate to severe, 0.5–0.6 as severe, 0.6 to 0.7 as severe to extreme, and greater than 0.7 m^2/3^ s^−1^ as extreme, and these thresholds were used in the official GTG 2.5 evaluation (Wandishin et al. 2011). Since commercial aircraft typically fly at airspeeds near 250 m s^−1^, a 1-minute flight segment may be 15 km in length, and the peak EDR locations may be in error by as much as 8 km or so. Nevertheless, these uncertainties are significantly less than those of pilot reports (PIREPs), which have traditionally been used for turbulence forecast training and evaluation. PIREPs are subjective, non-representative and frequently involve significant errors in the reported event’s location and time (Schwartz 1996). The high temporal and spatial resolution and objective nature of the *in situ* EDR reports make them ideal for the present study.

### Predictor data

#### Numerical weather prediction model and diagnostics

Despite their limitations, NWP model data and derived diagnostics provide valuable information about the storm environment as well as turbulence produced by large-scale, long-lasting forcing mechanisms. The DCIT training set includes 2-D and 3-D fields derived from the 13-km WRF-RAP NWP model including winds, turbulent kinetic energy (TKE), convective available potential energy (CAPE), convective inhibition (CIN), potential temperature, humidity mixing ratio, and many others. It also makes use of derived quantities and diagnostics that have been developed for turbulence forecasting including Richardson number (Ri), structure function eddy dissipation rate, horizontal and vertical shear, inverse stability, tropopause height, tropopause strength and a large number of others (Sharman et al. 2006b). In addition, based on studies like those described in Lane et al. (2012) and intuition about possibly relevant environmental factors, a number of new quantities were computed, e.g., distance above or below the tropopause.

#### Ground-based Doppler radar

The nation’s network of Weather Surveillance Radar-1988 Doppler (WSR-88D) radars, also known as NEXRADs, provides information about the location and intensity of clouds and storms. The DCIT training set includes information about the radar echoes, known as radar reflectivity, provided by the National Severe Storms Laboratory (NSSL) National Mosaic system (Zhang et al. 2011). These include composite reflectivity (the maximum reflectivity in a column), echo tops (the highest altitude with reflectivity ≥18 dBZ), and vertically-integrated liquid (an estimate of the total liquid water in a column).

Additionally, the Doppler capabilities of the radars can be used to measure wind variability and thereby determine turbulence in clouds where the radar signal is sufficiently strong and free of contaminants. The NTDA (Williams et al. 2006, 2008b) computes EDR from individual radar sweeps and merges the results from 133 NEXRADs over the CONUS to produce 3-D maps of in-cloud turbulence. Note that the NTDA is not able to detect turbulence outside the cloud boundary, which must be inferred by other means. In addition to the 3-D EDR field, 2-D turbulence “tops” fields were created from the highest altitudes of NTDA-detected light, moderate, and severe turbulence.

#### Geostationary satellite imagery

The National Oceanic and Atmospheric Administration maintains geostationary operational environmental satellites (GOES) that make periodic radiometric measurements of the earth at several channels of the electromagnetic spectrum with a temporal frequency ranging from 15 minutes over the CONUS to 3 hours for other parts of the hemisphere. These include visible light, channel 2 (3.9 μm wavelength), channel 3 (6.7 μm), channel 4 (10.7 μm), and channel 6 (13.3 μm) windows that provide information about clouds and storms. In addition to the raw imager data, several derived products were computed. For example, overshooting tops, identified using the longwave infrared 10.7 μm channel (Bedka et al. 2010), are satellite signatures of convective cells that penetrate into the lower stratosphere and indicate the likely generation of gravity waves. The presence of a tropopause fold, where the tropopause changes rapidly in height, is often associated with turbulence and may be derived using the water vapor (6.7 μm) channel (Wimmers and Moody 2004; Wimmers and Feltz 2012).

#### Lightning detection network

Lightning strike data from the National Lightning Detection Network (NLDN) owned by Vaisala Oyj (Cummins and Murphy 2009) is also used. The number and frequency of lightning strikes are often related to the intensity of the storm and the updraft inside it. In fact, Deierling et al. (2011) show evidence that lightning density may be related to the volume of in-cloud convective turbulence as measured by the NTDA.

#### Derived fields and features

In order to investigate the predictive value of the observed and model quantities described above, a number of derived fields were computed via local neighborhood filters, arithmetic combinations of fields, and distances to certain thresholds. For example, to capture information about spatial scales and proximities, statistics such as max, min, mean, standard deviation and number of good measurement points were computed over 10, 20, 40, 80 and 160-km radius discs for the radar composite reflectivity, composite reflectivity height and echo tops fields, the NTDA turbulence tops fields, the satellite imager fields and the 3-D NTDA EDR and reflectivity fields. Vertical distances were computed from the aircraft altitude to various reflectivity and EDR thresholds, to the tropopause, and to the surface. Differences were computed between the temperature at the aircraft altitude or the surface and various statistics of the GOES 10.7 μm brightness temperature, which represents the temperature of the cloud top or, when no cloud is present, the Earth’s surface. Differences were also computed between the various GOES channels, and between values of their disc statistics. Such differences can often be powerful predictors; for instance, the difference between the longwave infrared (10.7 μm) and water vapor (6.7 μm) channels was found so effective at determining thunderstorm intensity that it has been named the Global Convective Diagnostic (GCD, Martin et al. 2008). Finally, distances were computed from the aircraft *in situ* EDR report location to various features including reflectivity, NTDA, lightning density and echo top contours, overshooting tops, and tropopause folds. In order to determine whether direction was important, these distances were also computed within six 60^∘^ “wedges” oriented relative to the wind vector at the aircraft location. In all, 1030 candidate predictors were computed for this investigation. However, the results presented here do not utilize the individual wedge distances, leaving a total of 778 candidate predictors.

### Dataset preparation

#### Preparing “truth” data

A ground-based quality control algorithm monitors the distribution of the *in situ* EDR reports from each aircraft and flags them as bad if it deviates from expected norms. This happens periodically, and may result from a software, communications or instrument malfunction. The archived turbulence data are sorted into flights, and omitted data positions and times are estimated via interpolation. For DAL data, if routine reports are present every 15 minutes, intermediate reports are assumed to be missing because the reporting threshold of 0.2 m^2/3^ s^−1^ is not met; 14 reports of zero turbulence are inserted into these segments. (Note that the 0.0–0.2 m^2/3^ s^−1^ interval therefore has the correct total count, but not the correct distribution within that interval.) The time, distance and change in altitude of each flight segment between reports is computed, and only reports associated with time intervals between 45 and 75 seconds and altitude changes less than 3,000 ft are kept in the dataset. The position used for collocation with predictor variables is computed as the midpoint of each one-minute flight segment.

#### Handling missing predictor data

Predictor data can be missing for many reasons. For instance, an NWP model run or measurement may be missing from the archive maintained at NCAR or may be corrupted. Radar data are missing if the aircraft report is out of radar range or if there was no weather to return a signal. Data values that are missing for a known reason (e.g., no weather present) are replaced with an appropriate substitute value or flag. Instances for which one or more of the predictor variables is missing for an unknown reason are omitted from the dataset. While imputation or random assignment can be used to deal with missing data, eliminating compromised instances ensures the integrity of the dataset and facilitates the comparison of several statistical learning methods. For the present study, which utilizes data collected between March 10 and November 4, 2010, this procedure reduced the DAL dataset from 7,964,159 to 5,623,738 and the UAL dataset from 10,583,369 to 6,595,922 instances.

## Machine learning technique: random forest

The machine learning method used in this study is the random forest classifier (Breiman 2001). Random forests (RFs) are collections of weak, weakly-correlated decision trees that function as “ensembles of experts.” Each tree is trained using a bootstrap sample of the training data, and at each node the best split is selected from among a random subset of the predictor variables. This process ensures that each tree utilizes the training data and predictor variables in a different way, reducing its statistical dependence on the other trees. RFs have seen numerous successful applications over the past decade, particularly in the biomedical field (e.g., Díaz-Uriarte and de Andrés 2006). They have also been successfully used in a number of environmental science applications. For example, Pal (2005) showed that an RF could improve satellite radiometer-based land cover classification and argued that it was simpler to use than alternatives such as support vector machines. RFs have also been used by the author for thunderstorm prediction (e.g., Williams et al. 2008a) and for earlier work on CIT diagnosis (e.g., Williams et al. 2008b).

RF appears to be a good candidate methodology for diagnosing CIT using the diverse set of predictors described above. Many, such as the GTG diagnostics, are monotonically related to turbulence likelihood or intensity, but others may have a more complicated predictive relationship or are useful primarily in concert with other variables. The RF has the potential to utilize the joint distribution of the predictor variables. Used for classification, the votes of the RF can be related to a probability of turbulence. And RFs are known to be less susceptible to overfitting a training dataset, making them more likely to generalize well without the need for “optimal stopping” or similar techniques.

Moreover, because not all training instances are used to train each decision tree, those not used—the so-called out-of-bag instances—may be used to evaluate the performance of that tree, and this provides a way of quantifying the “importance” of each predictor variable. The RF estimates the “permutation accuracy importance” for each predictor by computing the degradation in classification performance over out-of-bag instances for each tree when the predictor’s values are permuted over instances, then aggregating the results. These importance assessments can be quite useful in selecting predictors from a large set of candidates, as described below.

## Empirical results

### Training and testing datasets

Because the main purpose of this study is to predict turbulence associated with convection, only *in situ* EDR reports within 80 km of rain regions (composite reflectivity ≥18 dBZ) were used. To help ensure independence between the training and testing datasets, training data were sampled only from odd Julian days and testing data only from even Julian days, or vice-versa. UAL and DAL datasets were analyzed separately because it wasn’t clear that their EDR values were identically calibrated. The data were also split into upper levels (above 25,000 ft) and mid-levels (10,000–25,000 ft); turbulence mechanisms are often different in these two levels, and the dataset includes many more *in situ* EDR reports at upper levels where aircraft cruise that would dominate a combined dataset. No UAL data below 20,000 ft were used because they are deemed unreliable (Larry Cornman, personal communication). The resulting distribution of EDR values is shown in Table 1. The RF was trained to use the collocated predictors described in Sect. 2, including the nearest observations in time and space and NWP model data interpolated to the aircraft position, to predict whether aircraft *in situ* peak EDR measurements would exceed 0.2 m^2/3^ s^−1^, the approximate threshold for light-to-moderate turbulence. This operationally significant turbulence is a rare event, constituting 1.33 % of DAL mid-level reports and just 0.25 % of DAL and UAL upper-level values. To address this imbalance, the RF training sets under-sampled the below-threshold instances to achieve good RF yes-no discrimination. The trained RFs were calibrated to provide both turbulence probability and deterministic EDR assessments.

**Table 1:** Counts and percentages of *in situ* EDR reports within 80 km of rain for mid and upper levels

In situ EDR (m^2/3^ s^−1^) range	Turb. category descriptor	DAL, mid-level		DAL, upper-level		UAL, upper-level	
		Count	Percent	Count	Percent	Count	Percent
0.0 to 0.1	*null*	347,794	94.956 %	1,707,998	98.583 %	2,167,839	97.759 %
0.1 to 0.2	*light*	13,605	3.7145 %	20,252	1.1689 %	44,193	1.9929 %
0.2 to 0.3	*light-mod*.	4,057	1.1077 %	3,670	0.2118 %	4,497	0.2028 %
0.3 to 0.4	*moderate*	678	0.1851 %	513	0.0296 %	766	0.0345 %
0.4 to 0.5	*mod*.*-sev*.	118	0.0322 %	100	0.0058 %	161	0.0073 %
0.5 to 0.6	*severe*	15	0.0041 %	11	0.0006 %	57	0.0026 %
≥0.6	*sev*.*-extr*.	1	0.0003 %	7	0.0004 %	31	0.0014 %
Total		366,268	100.000 %	1,732,551	100.000 %	2,217,544	100.000 %

### Performance evaluation

In order to be approved for operational deployment, GTG 2.5 underwent an independent evaluation (Wandishin et al. 2011) in which turbulence “events” derived from PIREPs and *in situ* EDR measurements were compared to the maximum GTG value within the neighborhood of a report. Statistics were computed for probability of detection (PODy), probability of false detection (PODn) for light, moderate, and severe turbulence thresholds, and receiver operating characteristic (ROC) curves were produced. ROC curves plot PODy vs. 1−PODn as the prediction threshold varies; the closer the curve comes to the upper left corner (PODy and PODn both 1), the closer the prediction is to perfect. GTG performance was also compared to AIRMETs, showing a comparable PODy but smaller area covered, a positive result.

In the present study, only *in situ* EDR reports are used as “truth” since they are objective measurements and have much more accurate positions and timestamps than PIREPs—an essential feature for capturing small-scale, dynamic CIT. Individual reports are used rather than multi-report “events” in order to allow calibration of RF vote counts into probability of a turbulence encounter in a 1-min flight segment. The area under the ROC curve (AUC) provides a valuable performance measure. As an integral, it is a stable statistic. Moreover, it is insensitive to the relative numbers of events and non-events, meaning that it is minimally affected by pilots’ having information that allows them to avoid many turbulent regions. And it is closely related to the ROC curve, which allows users to select thresholds to control the tradeoff between PODy and false alert ratio (FAR, 1−PODn). In addition to AUC, performance was evaluated by taking the maximum true skill statistic (TSS) and critical success index (CSI) over all the prediction thresholds, thus providing additional threshold-independent evaluation metrics. Here TSS, which is also known as the Hanssen-Kuipers Discriminant, is equal to PODy+PODn−1 and is thus a useful summary of overall prediction accuracy. CSI is the ratio of correct prediction “hits” to the sum hits + misses + false alerts; since it ignores correct nulls, CSI is often used to evaluate forecasts of rare events such as severe thunderstorms (e.g., Schaefer 1990), and so is ideal for evaluating turbulence predictions.

The actual utility of improved turbulence predictions will vary based on the end-user (e.g., airline dispatcher, GA pilot, or air traffic controller), the scenario (e.g., air traffic congestion, time of day), the spatial and temporal structures of the turbulent regions, and other factors. Thus, while an aggregate metric that takes into account all the economic costs and benefits over the U.S. air transportation system might be desirable, creating a comprehensive metric would be difficult and it would be complicated to use in practice. However, performance evaluations like ROC curves could be used by users to set their own thresholds based on the relative costs of false positives and false negatives in the context of their operational decisions. For example, Williams (2009) showed how to compute optimal routes given probabilistic aviation weather forecasts and a financial model for fuel use and the average cost of a hazardous weather encounter.

### Variable selection

The permutation accuracy importance estimates produced by an RF during training are based on classification accuracy, not contributions to the final probabilistic or deterministic predictions, and it has been shown that they can be biased when the predictor fields have varying scales of measurement (Strobl et al. 2007), as is true for this dataset. Nevertheless, predictors that have very low or zero RF importance seem unlikely to contribute much to a final model; thus, RF importance may be used to whittle down the list of candidate predictors. Predictor importances were evaluated using separate RFs for UAL and DAL, upper and mid-levels, and even and odd Julian days. The lowest performing predictors were removed, particularly if they appeared to duplicate the kind of information contained in higher performers or if they were particularly expensive to compute. Through this process, the list of candidate predictors was reduced to 107.

A second stage of the variable selection process was performed via a set of forward and backward selection experiments using AUC as the objective function. For each experiment, random subsets were drawn from the training and testing datasets. Two steps of forward selection (a pass through the unselected candidates to find which would yield the best performance when added to the predictor list) were followed by one step of backward selection (a pass through the predictors to see which could be removed with the least impact on performance). This procedure was performed for DAL and UAL, upper and mid-levels, and with even and odd Julian days used for the training dataset. A split of 70/30 non-turbulence to turbulence cases was selected through sensitivity tests with only 50 RF trees. The experiments were also performed using logistic regression. With this small number of trees, RFs did not perform statistically better than logistic regression on AUC or max TSS, but did obtain a slightly better max CSI. The cross-validation performance reaches a maximum after 50 to 60 iterations, at which point 17–20 predictors have been selected.

Table 2 shows the top 20 predictors for predicting DAL EDR≥0.2 m^2/3^ s^−1^ at upper levels using RF (left) and logistic regression (right) based on their average occurrences over 8 forward/backward selection experiments of 150 iterations each. Both results show a mix of model, satellite, and radar-derived fields, suggesting that these sources provide complementary information. The predictors selected by logistic regression are all monotonically related to the likelihood of turbulence. On the other hand, the RF results include a surprise, 160-km mean of Satellite Channel 6 (rank 8), which hadn’t previously been known to be related to CIT, plus plausible predictors not monotonically related to turbulence, including NWP model atmospheric pressure (rank 12), which is related to altitude. These fields may have non-linear relationships to turbulence, or may modulate the predictive relationships of the other variables. In any case, it appears that RF may have the potential to discover and exploit more complex relationships. This hypothesis is supported by the RF’s superior performance, as described in the following section.

**Table 2:** Forward/backward selection procedure results for RF and logistic regression

Random forest (8)			Logistic regression (8)		
Rank	Mean occ.	Predictor name	Rank	Mean occ.	Predictor name
1	115	Dist. to NSSL echo top >10 kft	1	135	Model FRNTGTHRI
2	114	Model FRNTGTHRI	2	134	Diff. Alt. to 80-km max NTDA sev. top
3	104	Model RITW	3	127	Dist. to NSSL echo top >10 kft
4	89	Model ELLROD2	4	126	10-km max of NSSL echo top
5	88	Diff. Alt. to 80-km max NTDA sev. top	5	121	Model ELLROD2
6	85	Model MWT2	6	111	Model RITW
7	79	Model ELLROD1	7	107	Model BROWN2
8	78	160-km mean of Satellite Ch. 6	8	94	Diff. Alt. to 20-km max NTDA mod. top
9	69	Model F2DTW	9	92	Model BROWN1
10	68	Model MWT3	10	89	Model ELLROD1
11	68	40-km min of Satellite Ch. 6	11	88	Model MWT3
12	67	Model Atm. Pressure	12	87	Model EDRRI
13	66	Model BROWN2	13	85	Model DTF3
14	65	Satellite Ch. 4 minus Model temp.	14	83	20-km no. of good NTDA dBZ points
15	64	Model DUTTON	15	77	10-km no. of good NTDA dBZ points
16	63	Satellite Ch. 4 minus Satellite Ch. 3	16	76	10-km mean of NTDA composite EDR
17	58	Model NGM2	17	74	10-km max of NTDA composite EDR
18	56	160-km mean of Satellite Ch. 4	18	74	10-km min of Satellite Ch. 3
19	53	Model RICH	19	73	10-km mean of NSSL echo top
20	52	Diff. Model pres. to Mod. surf. pres.	20	69	Model IAWINDRI

### Evaluation and calibration

**Fig. 1:**
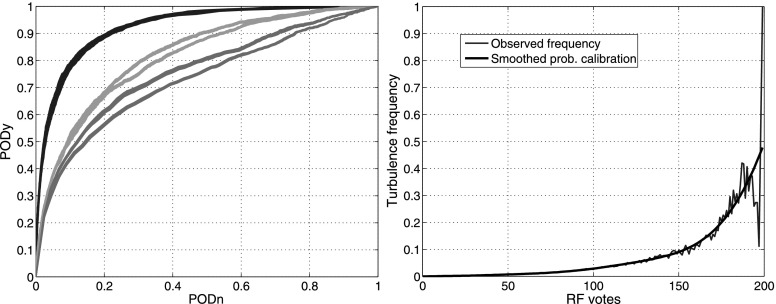
(*Left*) ROC curves from the 32 DAL upper-level cross-validation experiments for the RF (*blue*), GTG 3 prototype (*green*), and distance to storms as indicated by NSSL echo top>10,000 ft (*magenta*). (*Right*) Votes to probability calibration curve for the DAL upper-level RF (Color figure online)

The variable selection process ultimately resulted in identification of 39 predictors that contributed significantly to one or more of the upper or lower-level DAL or UAL datasets. These were used to perform 32 cross-validation experiments, training RFs on samples from even Julian days and testing them on samples from odd Julian days and vice-versa, using a 90/10 split that sensitivity studies found ideal for 200 trees. For comparison, identical cross-validation experiments were performed using *k*-nearest neighbors (KNN) with *k*=100 and logistic regression (LR); the skill of the GTG 3 1-hr forecast and “storm distance” were also evaluated. Storm distance was defined as the distance to NSSL echo tops exceeding 10,000 ft, i.e., the top predictor in the RF variable selection (Table 2) and the best of several observation-derived fields that were evaluated. Table 3 shows the skill statistics including AUC, max CSI, and max TSS for each of these. Figure 1 (left) shows the ROC curves for all 32 cross-validation experiments. The RF ROC curves are quite stable, but those for GTG 3 and storm distance vary based on whether the testing dataset is from odd or even Julian days, yielding two distinct sets of curves. The ROC curves and statistics show that the RF provides better performance in diagnosing CIT than the other methods. Note that the prototype GTG 3 was tuned using PIREPs and EDR data collected over 2010 and 2011, so this is not an unfair comparison. Also, although the variable selection process utilized both training and testing sets, the danger of overtraining is minimal because numerous selection experiments were aggregated, the number of selected predictors is relatively large, and the cross-validation experiments were performed independently.

**Table 3:** Skill score comparisons for DAL upper levels (*top*) and lower levels (*bottom*), including standard deviation, over 32 cross-validation experiments

DAL upper level skill scores (32)						
Method	AUC	Std	MaxCSI	Std	MaxTSS	Std
RF	0.924	0.002	0.075	0.006	0.699	0.006
KNN	0.915	0.001	0.064	0.003	0.688	0.003
LR	0.915	0.004	0.060	0.005	0.677	0.011
GTG 3	0.816	0.008	0.034	0.002	0.483	0.011
Storm distance	0.743	0.016	0.021	0.0005	0.389	0.026

DAL lower level skill scores (32)						
Method	AUC	Std	MaxCSI	Std	MaxTSS	Std
RF	0.911	0.002	0.137	0.005	0.678	0.004
KNN	0.895	0.002	0.114	0.005	0.645	0.004
LR	0.893	0.002	0.112	0.003	0.638	0.004
GTG 3	0.736	0.005	0.069	0.002	0.377	0.011
Storm distance	0.650	0.009	0.042	0.003	0.243	0.009

A final RF was created using the same sample size but drawing training data from all days. Its vote counts were calibrated into probabilities by combining the test set results from the 32 cross-validation experiments and tallying the frequency of turbulence observed for each vote count. Figure 1 (right) shows the raw frequencies and a smoothed calibration curve. The maximum observed frequency is under 100 %; this is not surprising since turbulence is an essentially random process, and predicting turbulence encounters with certainty at 1-min resolution is very difficult. Additionally, the training dataset includes turbulence reports only where aircraft fly, which rarely include places where turbulence is very likely. The RF votes were also calibrated to EDR by mapping the votes to the lognormal distribution of aircraft peak EDR measurements. Since the mappings are monotonic, the estimated AUC, max TSS and max CSI for both calibrated products are the same as those for the vote-based cross-validation experiments.

The selection of training and testing sets from odd and even Julian days is intended to produce representative yet independent sets (e.g., if instances were drawn completely randomly, adjacent turbulence reports might often end up in different sets), and the cross-validation estimates how well an RF will generalize. To test the accuracy of this approach, data from March 13–September 15 of both 2010 and 2011 were assembled, though NTDA fields weren’t available in 2011 and so were omitted. Using DAL upper-level data, 32× cross-validation with training and testing datasets drawn from odd and even Julian days produced the following skill scores and (standard deviations): mean AUC=0.919(0.002), MaxCSI=0.056(0.005), and MaxTSS=0.695(0.006). An identical cross-validation experiment with training and testing dataset pairs drawn from 2010 and 2011 produced a mean AUC=0.915(0.009), MaxCSI=0.051(0.005), and MaxTSS=0.688(0.019). Thus, the RF’s generalization between years was slightly poorer than suggested by cross-validation using odd and even Julian days; however, this difference was small relative to the disparity with the GTG 3 1-hr forecast scores: mean AUC=0.795(0.004), MaxCSI=0.027(0.001), and MaxTSS=0.449(0.005). DCIT’s skill will also be affected in practice by the lag time between its creation and subsequent use over the next 15 minutes of evolving weather before the next diagnosis. However, there are also time differences between the predictor field valid times and the *in situ* EDR reports used for the cross-validation study, and case study time loops of the RF-based DCIT turbulence diagnoses generally show smooth transitions from one frame to the next. Thus, while it will be fully considered in the official GTG 3/GTGN evaluation, the lag isn’t expected to significantly erode the superiority of DCIT over the valid GTG forecast.

### Case study

In the evening of May 25, 2011, a line of deep convective storms developed from eastern Texas to western New York and significantly disrupted air traffic by blocking most east-west routes. The FAA’s Air Traffic Control System Command Center database, available online at www.fly.faa.gov, indicates that at 00:24 UTC on May 26, 2011, an advisory was issued noting constraints due to weather in the Air Route Traffic Control Center (ARTCC) areas for KZOB (Cleveland), KZID (Indianapolis), and KZME (Memphis) until 04:30 UTC that required re-routes and “metering” of flights. Constraints were already in effect for the KZKC (Kansas City) and KZAU (Chicago) ARTCCs, and air traffic involving nearly all major U.S. airports was affected. Ground delay programs were in effect at several airports including Detroit beginning at 00:04 and Memphis at 01:07, and ground stops were ordered at Chicago O’Hare at 01:48, Indianapolis at 2:01, and Louisville, KY at 02:45 UTC. Delays mounted rapidly. Despite the unusually disruptive weather, the NWP model-based GTG forecast did not predict any significant turbulence near the line of storms, and pilots, dispatchers, and air traffic managers were left to use heuristics, SIGMET polygons, and the FAA’s storm avoidance guidelines to assess the hazard and make decisions about flight cancellations, delays, and re-routes.

**Fig. 2:**
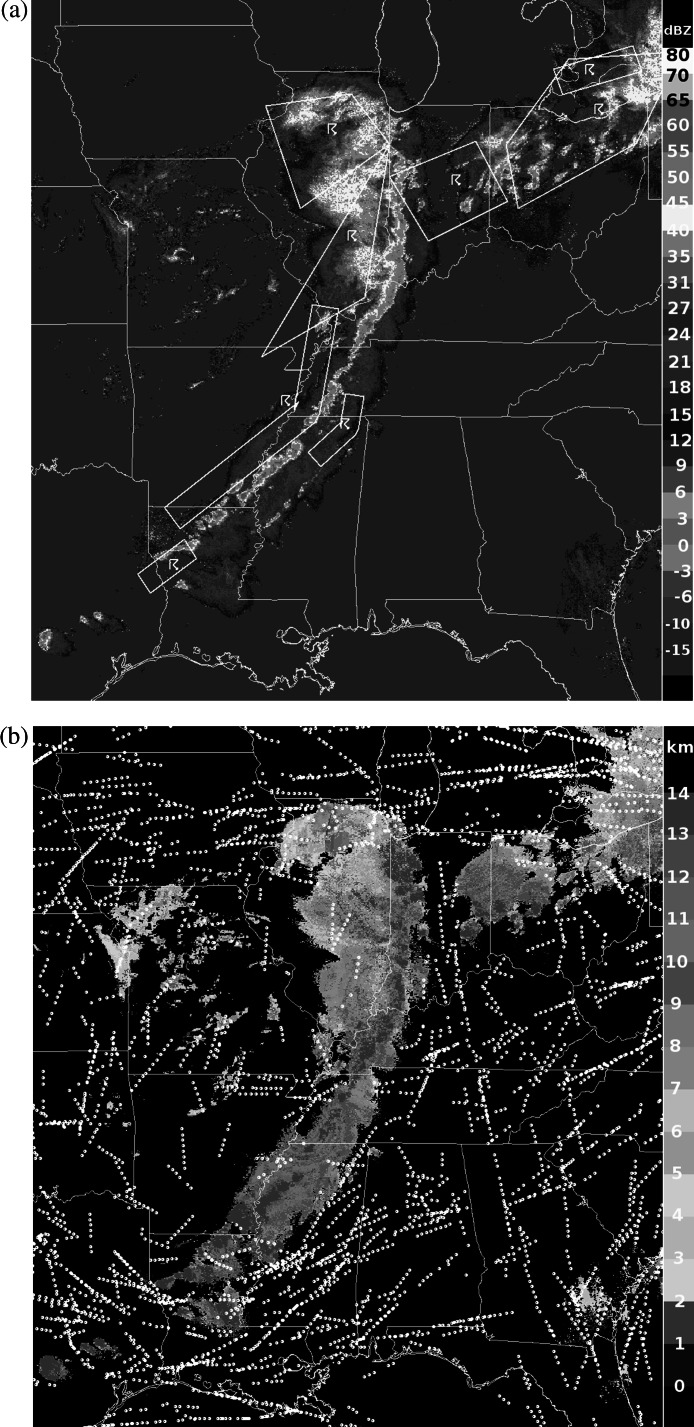

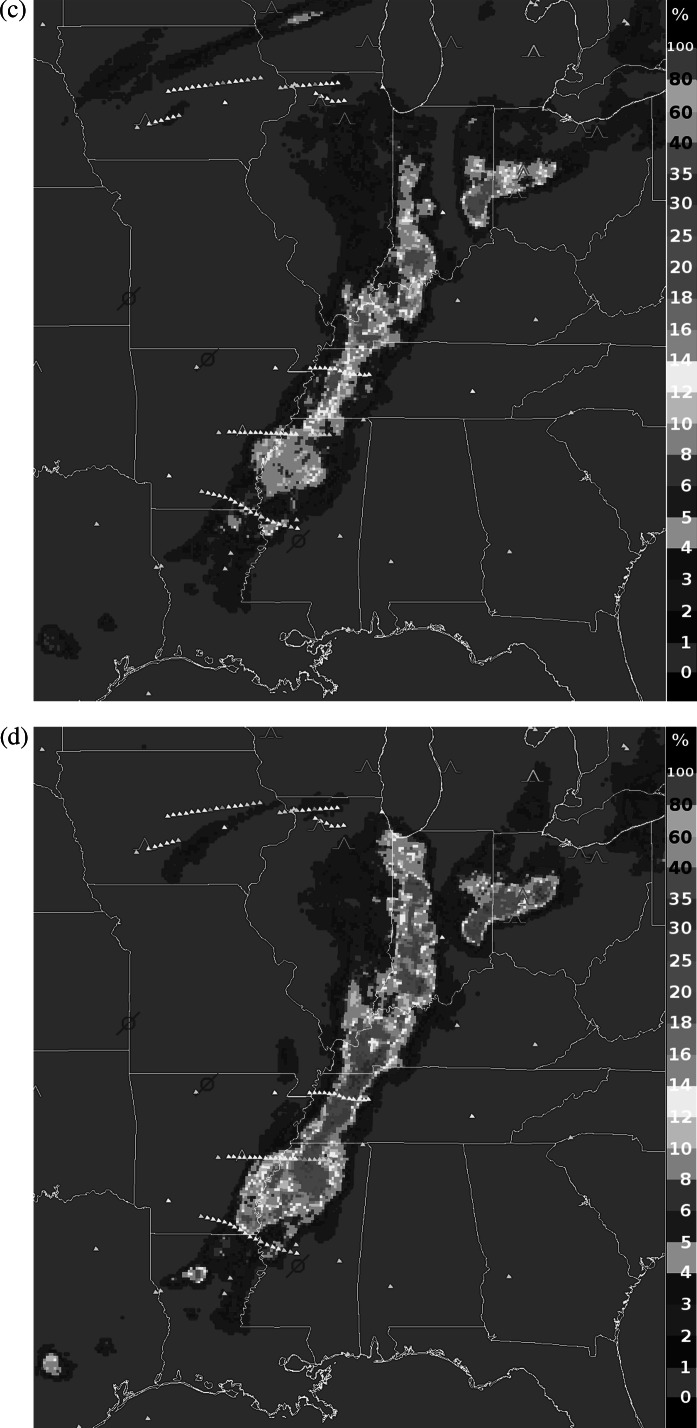
Case study from May 26, 2011 0200 UTC (evening of May 25 local time). (**a**) NSSL composite reflectivity (−15 to 60 dBZ), with SIGMETs overlaid; (**b**) NSSL echo tops (0 to 14 km), with ASDI aircraft positions overlaid; (**c**) DCIT turbulence probability (0 to 100 %) at FL330, about 33,000 ft, with PIREPs and DAL EDR reports overlaid; (**d**) DCIT probability (0 to 100 %) at FL390, about 39,000 ft, with PIREPs and DAL EDR reports overlaid (Color figure online)

Figure 2 illustrates this case. Figure 2(a) shows composite reflectivity (dBZ) with SIGMETs issued at 23:00 UTC on May 25 and valid until 01:55 UTC on May 26 overlaid. Figure 2(b) displays NSSL echo tops (km), with Aircraft Situation Display to Industry (ASDI) aircraft position reports between flight level 300 (FL300) and FL400 (about 30,000 to 40,000 ft) and 01:30 to 02:00 UTC overlaid. The SIGMETs and ASDI data confirm the severe impact of the storm system. On a normal day, the majority of flights are on east-west routes, but the ASDI tracks show pilots mostly flying north or south to skirt the storm, and only a few attempting penetrations. Figures 2(c) and 2(d) show DCIT RF turbulence probabilities from 01:45 UTC at FL330, about 33,000 ft, and at FL390, about 39,000 ft, with PIREPs and DAL *in situ* EDR reports overlaid. PIREPs are displayed as yellow, orange or red inverted V’s or blue null symbols. The upper-level PIREPs in the storm area of interest include moderate chop in northern Ohio, 01:51 UTC at 29,000 ft in a region where DCIT=4 %; moderate to severe turbulence in Western Ohio, 01:27 UTC at 38,000 ft where DCIT>20 %; and null turbulence in southern Kentucky, 01:52 UTC at 40,000 ft where DCIT=0. DAL *in situ* EDR reports from between FL300 and FL400 and 01:30 to 02:00 UTC are displayed as small triangles color-scaled from blue (null) to red (severe) turbulence. They include routine reports of null turbulence (EDR at most 0.06 m^2/3^ s^−1^) from six flights just to the east of the line of storms where DCIT=0, and one over Indiana with EDR=0.1 m^2/3^ s^−1^ at 37,000 ft where DCIT=4 %. Three east-west penetrations of the line of storms generated triggered DAL report sequences; they included, from north to south: (1) EDR=0.22 and 0.24 m^2/3^ s^−1^ at 36,000 ft where DCIT>20 %; (2) two reports of EDR=0.28 m^2/3^ s^−1^ at 36,000 ft where DCIT>20 %; and (3) one report of EDR=0.2 m^2/3^ s^−1^ at 37,000 ft where DCIT=5 %. A fourth east-west transit to the south that grazed Louisiana had two routine reports of EDR=0.02 m^2/3^ s^−1^ where DCIT=0 and one EDR=0.04 m^2/3^ s^−1^ at 36,000 ft where DCIT=2 %. The turbulence reports were well correlated with the DCIT diagnosis for this case.

The plots in Fig. 2 show that areas of enhanced DCIT turbulence probabilities are clearly related to elevated composite reflectivity or echo tops, but not coincident or correlated with them, and they also offer a guide to likely turbulence in clear air around the storms. On the other hand, an RF trained using only WRF-RAP predictor fields (not shown) had at most 1 % probability of turbulence over the entire storm area, suggesting that radar and satellite observations were essential ingredients for diagnosing the CIT associated with these powerful storms.

If the DCIT diagnosis had been available on this day via the GTGN product, it could have significantly aided commercial and general aviation pilots, airline dispatchers and flight planners who make use of the NWS AWC’s ADDS service, and might have supported AWC forecasters in keeping SIGMETs up to date. Rather than looking at several different model and observation sources of information, these users would have been able to directly utilize the fused assessments. For instance, pilots make it a priority to find the smoothest altitude for their flights, consistent with time and fuel usage requirements. The DCIT probabilities suggest that for aircraft forced to traverse the storm, the patchier turbulence at FL330 might have offered some safer penetration routes. This is particularly true around Chicago, where the DCIT prototype shows that the likelihood of turbulence is significantly reduced at FL330. Moreover, DCIT suggests a safe north-south route over central Indiana, which the ASDI data show that a few flights are taking. However, this region is blocked by a valid convective SIGMET that designates this area as unsafe, potentially limiting air traffic in an area that is actually benign.

## Operational infusion and anticipated benefits

An RF-based DCIT prototype is running in real-time at NCAR, producing gridded output at 6-km horizontal and 1,000 ft vertical resolution every 15 minutes for internal use in case studies, statistical performance evaluation, and integration into GTGN. The multi-threaded C++ implementation uses a decision tree to determine which RF and calibration to run based on altitude and on available predictor data; thus, it accommodates minor data outages gracefully. Input data are processed asynchronously as they arrive, including by advecting satellite, radar and lightning data forward to the next scheduled DCIT generation time to account for storm motion. This helps ensure that the data are temporally aligned and that latency will be minimal. Output is displayed graphically (as in Fig. 2) and analyzed to ensure its plausibility and spatial and temporal coherence. The DCIT EDR assessment is combined with the latest GTG 3 NWP-based turbulence forecast and “nudged” using recent PIREPs and *in situ* EDR reports to create the GTGN product. GTG 3, which includes GTGN as its nowcast component, is scheduled to be evaluated by the FAA AWRP’s Quality Assessment Product Development Team beginning in late 2013, likely using an approach similar to Wandishin et al. (2011). An FAA Technical Review Panel will review the evaluation, and if results are favorable, GTG 3 could be deployed for operational use at the NWS AWC as early as 2014. Users including pilots, airline dispatchers, aviation weather forecasters and private weather service providers will then be able to retrieve the gridded nowcasts for use in their own displays and automated systems or view them via the ADDS website introduced in Sect. 1.3.

DCIT’s deterministic EDR diagnoses will improve the operational aviation weather product, GTG, in the important domain of CIT assessment where it currently performs poorly, as has been shown via some of the same metrics used in official GTG evaluations. DCIT’s probabilistic assessments, which will be extended in the future to include short-term forecasts, will make an important contribution to the U.S. NextGen air traffic modernization effort (JPDO 2008) by providing authoritative information required for automated decision making (e.g., Krozel et al. 2007; Lindholm et al. 2010). For instance, Williams (2009) showed that dynamic programming could be used to choose optimal routes based on probabilistic forecasts. Quantifying DCIT’s impact on accidents, injuries, fuel costs, delays and cancellations will be difficult due to the complexity of the NAS and the diverse, decentralized, asynchronous decision-making processes involved at multiple timescales. However, it seems clear that, when fully integrated into NextGen, this improved information on CIT location and intensity will assist dispatchers and pilots in choosing efficient, safe routes near storms and will decrease the number of unexpected turbulence encounters and re-routing requests, reducing controller workload.

## Lessons learned

Using machine learning to diagnose turbulence requires dealing effectively with the rareness of encounters captured in the available “truth” data. Initial approaches to DCIT development using multi-category predictions or regressions were difficult to calibrate. Reducing the problem to binary classification facilitated rebalancing of the dataset and producing reliable probabilistic diagnoses, simplifying DCIT interpretation and optimization. It was found that the output of an RF trained to identify light-to-moderate or greater turbulence could be scaled to EDR, and it also performed well on discriminating moderate and severe turbulence (Williams et al. 2012). The use of a calibrated random forest classifier also facilitated the use of DAL data, whose use of “triggering” skews the distribution of small EDR values.

Domain knowledge was useful in selecting appropriate data sources, deriving features and transformations that better exposed the relevant information, and selecting “truth” data that adequately capture the temporal and spatial scales of the CIT phenomenon. RF importances provided a helpful guide to initial predictor selection but can be biased and do not expose correlations between variables. Using forward and backward selection appeared more reliable in identifying a minimal set of skillful predictors.

The RF did show better skill than logistic regression for this problem, perhaps because it makes better use of predictors not monotonically related to the predictand, and also better than *k*-nearest neighbors. It may be that some of the predictor variables could be transformed or combined to make them more effective in a simpler model, which would be preferable from the standpoint of computational intensity in a real-time system. This possibility will be further investigated. Other RF formulations (e.g., Menze et al. 2011) may also provide more useful variable importance information or improved performance. For instance, by utilizing object-based relationships rather than relying on the numerous neighborhood statistics employed in the present study, the Spatiotemporal Relational Random Forest (SRRF) approach described in McGovern et al. (2013) may provide a simpler, more easily interpreted and more flexible model.

In using a machine learning model in an operational system, it is important to minimize run-time while dealing gracefully with data latency and availability issues. The DCIT system was designed to handle the asynchronous arrival of real-time input data by immediately deriving features and performing forward motion-adjustment to align with the next product generation time. Predictor field alignment is critical; without it, RF output becomes “blurred” and less useful. Potential data outages are dealt with by utilizing similar or redundant sources of information and training different RFs for different data feed failure modes. An appropriate RF model is selected at run-time if sufficient input data are present to meet performance requirements.

A significant problem in applying empirically-based techniques for weather prediction is that the observation platforms and NWP models that supply predictor variables evolve over time, and weather phenomena themselves may vary based on climate change and large-scale patterns like the El Niño/La Niña–Southern Oscillation. During DCIT development, the training database was re-created after NOAA switched from the RUC to the WRF-RAP NWP model, and there were also changes in the radar and satellite systems. To perform optimally, DCIT will likely require periodically re-calibration after it becomes operational. Eventually, on-line training and calibration could be incorporated so that the system will automatically adjust to such changes.

In conclusion, as the amount of data from models and observations continues to grow at an exponential rate, techniques like the one described in this paper hold the promise of effectively exploiting the available data to provide societally relevant information.
